# Role of transcriptomics in the study of oral cancer

**DOI:** 10.3389/froh.2025.1524364

**Published:** 2025-07-28

**Authors:** Prabhu Manickam Natarajan, Vidhya Rekha Umapathy

**Affiliations:** ^1^ Department of Clinical Sciences, Center of Medical and Bio-allied Health Sciences and Research, College of Dentistry, Ajman University, Ajman, United Arab Emirates; ^2^ Department of Dentistry, Sri Lalithambigai Medical College and Hospital, Dr.M.G.R. Educational and Research Institute, Chennai, Tamilnadu, India

**Keywords:** oral cancer, transcriptomics, RNA sequencing, microarray analysis, small RNA profiling, biomarkers, heterogeneity, therapeutic targets

## Abstract

Oral cancer, a formidable public health challenge, demands innovative approaches for early detection and effective treatment. This review explores the transformative role of transcriptomics in oral cancer research, leveraging cutting-edge technologies to decode the RNA landscape of tumors. We delve into RNA sequencing (RNA-seq), single-cell RNA sequencing (scRNA-seq), long non-coding RNA sequencing (lncRNA-seq), microarray analysis, and small RNA profiling, showcasing their unique contributions to unraveling the complexities of oral cancer. By mapping gene expression patterns, identifying biomarkers, and understanding tumor heterogeneity at an unprecedented resolution, transcriptomics offers powerful insights into the molecular mechanisms driving oral cancer. Furthermore, we highlight how these approaches pave the way for personalized medicine, enabling precise therapeutic interventions and innovative treatment strategies. The review also addresses the future directions and challenges in the field, emphasizing the need for advanced computational tools and interdisciplinary collaboration to fully harness the potential of transcriptomics in transforming oral cancer care.

## 1 Introduction

Oral cancer represents a formidable global health challenge, characterized by the malignant proliferation of cells within the oral cavity, including the lips, tongue, gums, floor of the mouth, and inner lining of the cheeks (1). Despite advancements in diagnostic and therapeutic approaches, oral cancer continues to impose a significant burden worldwide, with escalating incidence rates observed across diverse populations. Information sourced from the Global Cancer Observatory (GCO) reveals that in the year 2020, oral squamous cell carcinoma (OSCC) had an annual global incidence of 377,713 cases. Asia reported the highest number of cases at 248,360, followed by Europe with 65,279 cases, and North America with 27,469 cases (2). The etiology of oral cancer is multifaceted, encompassing a complex interplay of environmental, behavioral, and genetic factors. Tobacco use, particularly smoking and smokeless tobacco consumption, stands as the predominant risk factor for oral cancer, contributing substantially to its global prevalence (3). Additionally, excessive alcohol consumption, betel quid chewing, human papillomavirus (HPV) infection, poor oral hygiene, dietary factors, and genetic predisposition further augment the risk of oral malignancies.

Clinically, oral cancer often manifests as painless ulcers or lesions in the oral cavity, frequently accompanied by symptoms such as dysphagia, persistent sore throat, hoarseness, and unexplained bleeding. Early detection and diagnosis are paramount for optimizing treatment outcomes, underscoring the critical importance of routine oral screenings, particularly among high-risk individuals (4). Treatment modalities for oral cancer typically encompass a multidisciplinary approach, integrating surgical intervention, radiation therapy, chemotherapy, and targeted therapies, tailored to the individual characteristics of the tumor and the patient.

In recent years, molecular profiling techniques have emerged as indispensable tools for unraveling the intricate molecular landscape of oral cancer. These techniques, including genomics, proteomics, epigenomics, metabolomics, and transcriptomics, offer unprecedented insights into the underlying pathogenic mechanisms, facilitate the identification of potential biomarkers for early detection and prognosis, and unveil novel therapeutic targets (5, 6). Among these, transcriptomics, which encompasses the comprehensive analysis of RNA transcripts produced by oral cancer cells, holds particular significance in elucidating the molecular intricacies of the disease. Through sophisticated methodologies such as RNA sequencing (RNA-seq) and microarray analysis, transcriptomic studies enable the identification of differentially expressed genes in oral cancer compared to normal tissues, thereby elucidating key pathways and biological processes implicated in disease progression6. Furthermore, transcriptomic profiling facilitates the discovery of biomarkers for early detection, prognostication, and prediction of treatment response in oral cancer patients.

**Figure 1:**
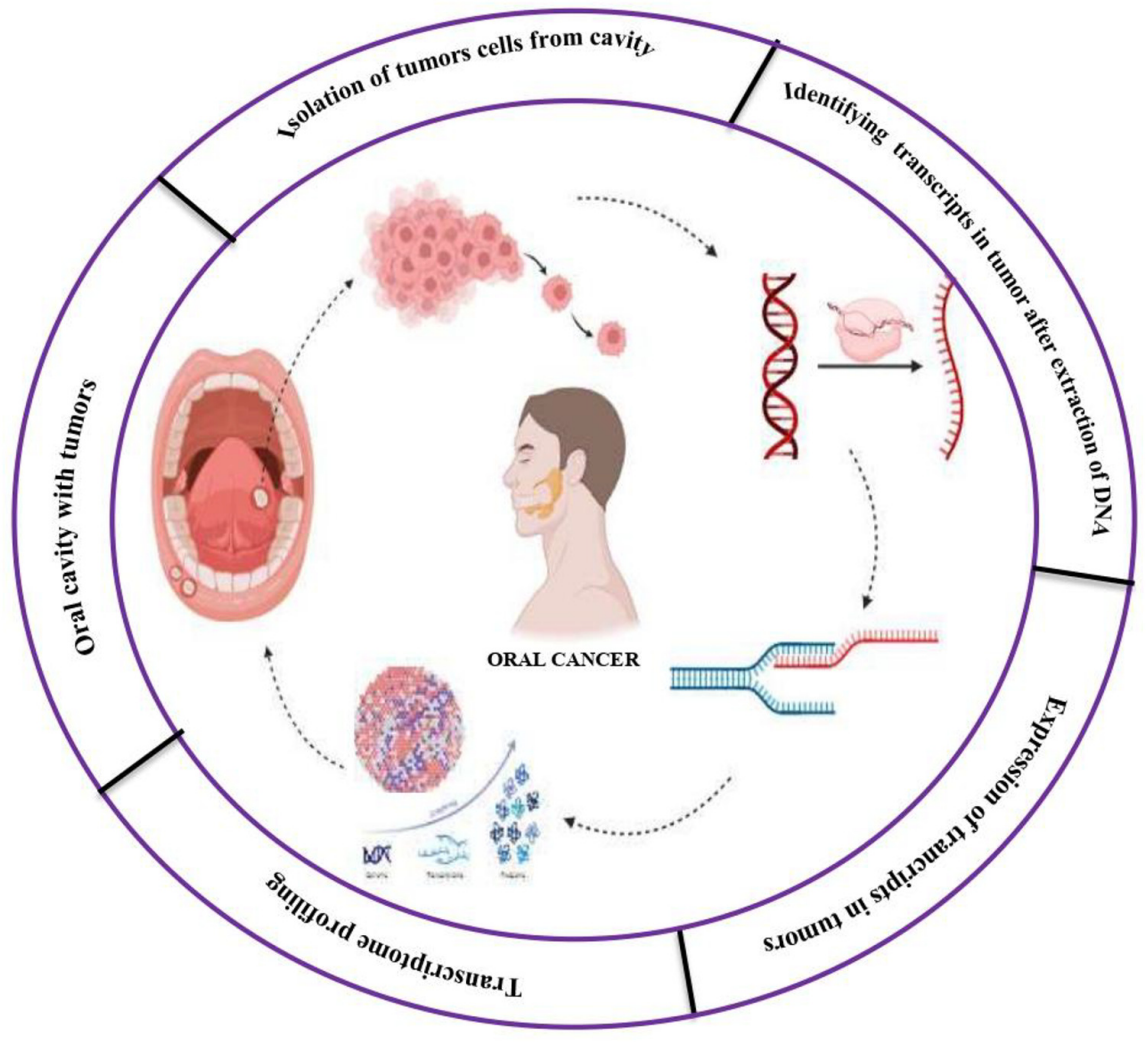
Transcriptomics in oral cancer.

Moreover, transcriptomic analyses contribute to our understanding of tumor heterogeneity, delineating the diverse cellular populations and transcriptional states within oral tumors. By comprehensively analyzing gene expression patterns and regulatory networks in oral cancer tissues, transcriptomic studies have revolutionized our comprehension of this complex disease, paving the way for personalized medicine approaches and enhanced patient outcomes. In this review, we explore the pivotal role of transcriptomics in oral cancer research, delineating its contributions to biomarker discovery, molecular characterization, treatment response prediction, and identification of therapeutic targets (Figure 1). Through an examination of the latest advancements and future directions in transcriptomic analysis, we aim to furnish a comprehensive overview of its implications for oral cancer diagnosis, treatment, and management.

## 2 Overview of transcriptomics

**Figure 2:**
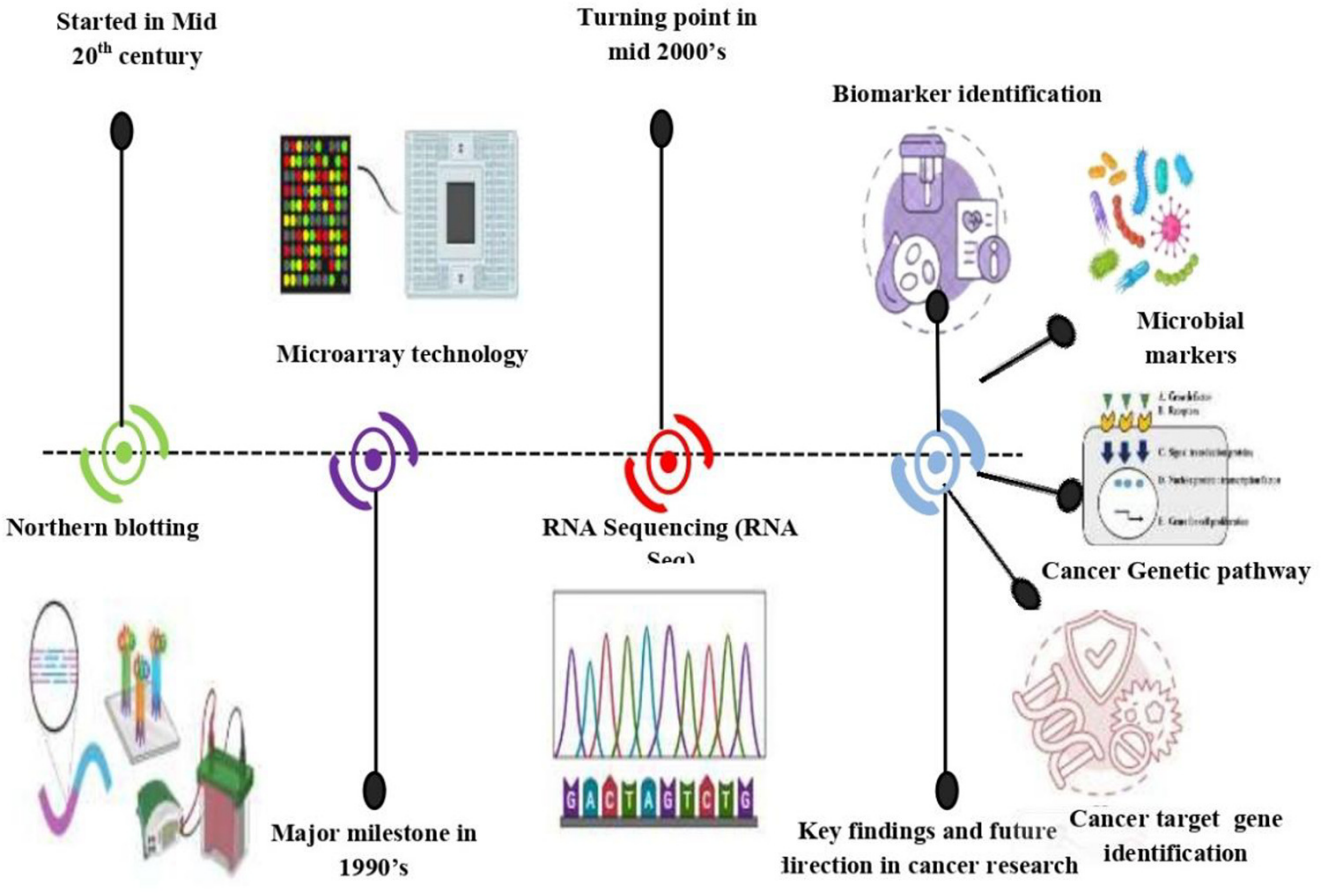
The transcriptomics landscape was described as follows: “Starting point”: commencement of transcriptomics via early gene expression studies like northern blotting. “Major Milestone”: Emergence of microarray technology in the 1990s, enabling genome-wide gene expression analysis. “Turning Point”: Revolutionary impact of RNA sequencing (RNA-Seq) in the 2000s, allowing precise RNA transcript characterization. “Key Findings and Advancements in Oral Cancer Research”: Significant discoveries such as biomarkers discoveries, microbial gene integration in cancer progression, disease gene target identification, genomic profiling, genetic pathway network prediction within oral cancer transcriptomics.

The term “transcript” refers to the RNA molecule synthesized from a DNA template during the process of transcription. It carries the genetic information encoded in the DNA and serves as a template for protein synthesis or performs various regulatory functions within the cell (7). On the other hand, “-omics” refers to the systematic study of a particular biological component or process on a comprehensive scale. It stems from the Greek word “omos,” meaning “whole” or “all.” The “-omics” suffix is commonly appended to various biological entities, such as genomes, proteomes, and metabolomes, to denote comprehensive analysis approaches aimed at understanding the entirety of these components within a biological system (8). The field of transcriptomics is a branch of molecular biology, emerged as researchers sought to comprehensively study the complete set of RNA transcripts produced by an organism, tissue, or cell, aiming to understand gene expression patterns and regulatory mechanisms on a global scale (9). Transcriptomics has its roots in the mid-20th century with the discovery of RNA's role in genetic processes (Figure 2). Early gene expression studies, such as Northern blotting, laid the groundwork. Microarray technology in the 1990s allowed for genome-wide gene expression analysis. Next-generation sequencing, notably RNA sequencing (RNA-seq), emerged in the 2000s, revolutionizing transcriptomic research. The term “transcriptomics” was coined to describe the systematic study of RNA transcripts (10). Transcriptomics has found extensive application in cancer research, enabling the profiling of gene expression patterns, identification of biomarkers, and elucidation of molecular mechanisms underlying tumorigenesis and treatment response. This journey reflects ongoing efforts to understand gene expression and regulation, shaping our understanding of biology and disease such as cancer, neurodegenerative disorders, and infectious diseases. Despite challenges like data analysis complexity and sample heterogeneity, ongoing advancements in transcriptomic technologies and computational methods promise to further enhance our understanding of gene expression dynamics and their implications for health and disease, shaping future research directions and clinical applications.

The application of transcriptomics in oral cancer research gained traction in the early 2000s, coinciding with the development of high-throughput sequencing technologies like RNA sequencing (RNA-seq) and microarray analysis. These methods allowed researchers to comprehensively profile gene expression patterns in oral cancer cells and tissues, providing insights into the molecular mechanisms driving tumorigenesis, tumor progression, and treatment response. Transcriptomic analysis in oral cancer encompasses the study of messenger RNA (mRNA), non-coding RNA (ncRNA), and other RNA molecules, offering valuable insights into gene expression patterns, transcriptional regulation, and RNA processing events within the context of oral tumors (11). At its core, transcriptomics aims to quantify and characterize RNA transcripts to understand how genes are expressed and regulated in oral cancer. Through transcriptomic analysis, researchers have identified dysregulated genes, signaling pathways, and molecular subtypes associated with oral cancer, paving the way for personalized medicine approaches and targeted therapies tailored to individual patients (12). Transcriptomics has become an integral component of oral cancer research, driving advancements in our understanding of the disease biology and the development of novel diagnostic and therapeutic strategies.

Primary techniques used in transcriptomics, such as RNA sequencing (RNA-Seq) and microarray analysis, have been instrumental in oral cancer research. RNA-Seq enables precise quantification and characterization of RNA transcripts in oral cancer tissues, allowing for the identification of gene expression changes, alternative splicing events, and novel transcripts, including non-coding RNAs (13). Microarray analysis, on the other hand, facilitates the simultaneous measurement of gene expression levels in oral cancer samples, enabling the detection of gene expression differences between normal and cancerous tissues (14).In addition to these primary techniques, secondary techniques used in transcriptomics, including Quantitative PCR (qPCR), *in situ* Hybridization (ISH), Reverse Transcription Polymerase Chain Reaction (RT-PCR), Ribosome Profiling (Ribo-seq), RNA Immunoprecipitation (RIP), and RNA Interference (RNAi), play crucial roles in validating transcriptomic findings and providing functional insights into gene expression and regulation in oral cancer. These techniques collectively contribute to a comprehensive understanding of the molecular landscape of oral cancer and drive the development of precision medicine approaches for improved diagnosis and treatment of the disease.

## 3 Transcriptomics approaches in oral cancer

Transcriptomic profiling techniques in oral cancer involve a variety of methods aimed at comprehensively analyzing gene expression patterns and regulatory networks within oral cancer tissues. These techniques enable researchers to identify dysregulated genes, molecular pathways, and potential biomarkers associated with oral carcinogenesis. There are Commonly employed transcriptomic profiling techniques in oral cancer research (Table 1).

**Table 1:** Comparison of transcriptomic techniques: overview of principles, advantages, and limitations of RNA sequencing (RNA-Seq), microarray analysis, quantitative PCR (qPCR), single-cell RNA sequencing (scRNA-Seq), long Non-coding RNA profiling, and small RNA profiling techniques in transcriptomic analysis.

Technique	Principle	Advantages	Limitations
RNA sequencing	Measures RNA abundance and identifies transcripts	High throughput, quantitative, detects novel transcripts	High cost, computational resources needed, data analysis complexity
Microarray analysis	Measures gene hybridization with probes	Cost-effective, multiplexed analysis, customizable	Limited dynamic range, requires prior knowledge of target genes expression using
Quantitative PCR	Measures gene expression using targeted amplification	Highly sensitive, specific, quantitative, high-throughput	Limited throughput, requires target-specific primers (qPCR)
Single-cell RNA	Measures RNA expression at single-cell level	Enables analysis of individual cells, detects rare cell populations	technical challenges such as low throughput, high cost Sequencing (scRNA-seq)
Long non-coding RNA	Profiling of non-coding RNA transcripts	Reveals regulatory roles of lncRNAs, potential biomarkers, disease associations	Technical complexity, limited annotations, requires specialized analysis tools for Profiling non-coding RNA
Small RNA profiling	Profiling of small RNA molecules (e.g., microRNAs, piRNAs)	Identifies microRNAs and other small RNAs, potential biomarkers, functional roles	Limited detection of low abundance RNAs, bias in library preparation RNA molecules.

### 3.1 RNA sequencing (RNA-SEQ)

RNA sequencing (RNA-Seq) has emerged as a cornerstone in elucidating the molecular intricacies of oral cancer, offering unparalleled resolution and depth in transcriptome analysis. It enables comprehensive profiling of gene expression patterns in both normal and cancerous oral tissues, facilitating the identification of crucial differentially expressed genes (DEGs) in oral cancer pathogenesis. The RNA-seq-based nomogram, as developed by Qiao et al. (15), provides a valuable tool for personalized treatment in oral squamous cell carcinoma (OSCC) patients, aiding in preoperative prediction of lymph node metastasis (LNM) by analyzing RNA-seq data from 276 OSCC patients.

Moreover, RNA-Seq allows for the discovery of novel transcripts, alternative splicing events, and fusion genes, shaping the transcriptomic diversity observed in oral cancer. Zhang et al. (16) explored the genetic landscape of oral squamous cell carcinomas (OSCCs) using whole transcriptome analysis, identifying significantly mutated genes (SMGs) and genes with disruptive mutations (TDGs) enriched in membrane-related functions and pathways like fibroblast growth factor (FGF) signaling and cell adhesion molecules (CAMs).Functional annotation and pathway analysis of RNA-Seq data unveil dysregulated signaling pathways implicated in oral cancer progression, providing mechanistic insights into disease pathophysiology. Khan et al. (17), analyzed the molecular profiles of potentially malignant lesions (PMLs) and OSCC through RNA sequencing, finding heterogeneity within PMLs and early activation of cancer-associated pathways in dysplastic samples.

Furthermore, RNA-Seq serves as a powerful platform for biomarker discovery, enabling the identification of molecular signatures indicative of disease diagnosis, prognosis, and therapeutic response. Tang et al. (18) analyzed RNA-seq data comparing early and late stages of tongue cancer development, offering insights for predicting cancer risk and staging tongue carcinogenesis. Whole Gene expression analysis will tell us about the interrelationship between the oral microbiota and the tumor progression. This associative approach was given by Khan et al. (17), their Findings suggest heightened gene expression in OSCCs and PMLs, particularly in pathways linked to epithelial-mesenchymal transition (EMT), KRAS signaling, and inflammatory processes. The study implies potential implications for the oral microbiome and tumor microenvironment through whole RNA sequencing platform.

Integration of RNA-Seq data with other omics datasets, including genomics, epigenomics, and proteomics, enables a multi-dimensional understanding of oral cancer biology. Studies like the one by Bohai Feng et al. (19), reveal genetic and epigenetic disparities, identify key regulatory genes like EGFR and PTGS2, and pave the way for precision medicine strategies tailored to individual patients, and pave way for improved promising treatment outcomes in oral cancer management. In essence, RNA-Seq stands as a cornerstone technology in advancing our understanding of oral cancer at the molecular level, offering unparalleled insights into its etiology, progression, and therapeutic vulnerabilities.

### 3.2 Microarray analysis

Microarray analysis has emerged as a cornerstone in unraveling the intricate molecular mechanisms underlying oral cancer, providing a holistic perspective on gene expression patterns and genomic alterations driving tumorigenesis. Zarzar et al. (20) showcased the power of microarrays in distinguishing oral dysplasia from squamous-cell carcinoma, employing innovative machine learning techniques to achieve remarkable classification accuracy. This study exemplifies the potential of microarrays in refining diagnostic approaches for oral cancer.

Furthermore, microarray studies have identified a plethora of differentially expressed genes (DEGs) implicated in critical cellular processes, shedding light on the underlying pathophysiology of oral cancer. Estilo et al. (21), utilized high-density oligonucleotide probe arrays to uncover gene expression disparities in oral tongue squamous cell carcinoma, laying the groundwork for comprehensive transcriptional profiling and prognostic assessment in this malignancy.

Ramaswamyreddy and Smitha (22) emphasized the significance of microarray analysis in elucidating the molecular events driving oral squamous cell carcinoma (OSCC) progression. By integrating clinical and molecular parameters, microarray analysis enhances our understanding of OSCC pathogenesis and facilitates more accurate risk assessment and management strategies, complementing traditional histopathological assessments. Moreover, microarray technology extends beyond gene expression profiling to uncover genomic alterations such as copy number variations (CNVs) and somatic mutations in oral cancer genomes. Integration of microarray data with other omics platforms, as demonstrated by Li et al. (23), offers a comprehensive understanding of the molecular landscape of oral cancer through an integrated omics approach, enabling the identification of novel biomarkers and therapeutic targets for precision medicine approaches.

Despite the advent of next-generation sequencing technologies, microarray analysis remains a valuable tool in the molecular characterization of oral cancer, providing complementary information and facilitating translational research efforts aimed at improving diagnosis, prognosis, and therapeutic interventions for patients afflicted with this malignancy. Patel et al. (24), analyzed gene expression in buccal mucosa squamous cell carcinoma (BMSCC) using microarray chips, They found 237 protein coding RNAs and 85 long non-coding RNAs, potential therapeutic targets and biomarkers, including EIF3J and SDCBP associated with survival outcomes.This underscores the enduring relevance and utility of microarrays in the era of precision oncology.

### 3.3 Quantitative PCR (qPCR)

In the landscape of transcriptomics, quantitative PCR (qPCR) emerges as a cornerstone approach, intricately linking gene expression patterns with the complex biology of oral cancer. Stephen et al. (25), unveil the epigenetic intricacies of oral squamous cell carcinoma (OSCC), quantitatively dissecting promoter hypermethylation levels in genes like uPA, PAI-1, and TIMP-1, shedding light on their pivotal roles in tumor progression. Mahnaz Fatahzadeh et al. (26) delve into telomerase mRNA expression, spotlighting its significance as a potential biomarker for diagnosing and prognosticating oral and oropharyngeal squamous cell carcinomas. Bouda et al. (27) scrutinize HPV-16 DNA levels in head and neck squamous cell carcinoma (HNSCC), elucidating its association with key genes like KAI1, E-cadherin, and beta-catenin, elucidating viral contributions to cancer etiology. Torabizadeh et al. (28) pioneer non-invasive detection of oncogenic HPV load in oral cancer, integrating qPCR insights with clinical diagnostics for enhanced patient management. Schubert et al. (29), offer nuanced biomarkers through quantitative protein expression analysis, highlighting genes intertwined with the transcriptomic landscape, enriching our comprehension of OSCC pathogenesis.

Lastly, Ohta et al. (30) unveil a gene-focused qRT-PCR assay, unraveling the intricate gene expressions of uPA, PAI-1, and TIMP-1 pivotal in OSCC progression and metastasis, epitomizing transcriptomic endeavors in deciphering oral cancer2 biology. These studies collectively illuminate the transcriptomic terrain of oral cancer, intertwining gene-centric qPCR analyses with molecular insights and therapeutic avenues.

### 3.4 Single-cell RNA sequencing (scRNA-SEQ)

Single-cell RNA sequencing (scRNA-Seq) has emerged as a transformative technology, enables the profiling of gene expression at the single-cell level enabling the characterization of individual tumor cells and their transcriptional profiles. By dissecting the transcriptomes of individual cells within the tumor microenvironment, scRNA-Seq offers insights into the diverse cellular populations comprising oral tumors, including cancer cells, stromal cells, immune cells, and endothelial cells. The study by Kurkalang et al. (31), provides unique insights into oral squamous cell carcinoma (OSCC) of the gingivo-buccal region (OSCC-GB) and its association with oral submucous fibrosis (OSMF). Utilizing scRNA-seq, the researchers identified specific malignant cell clusters, observed cellular reprogramming and epithelial-mesenchymal transition (EMT), and discovered antitumor T cell populations and mixed macrophage types. This study highlights the complexity of OSCC-GB and unveils potential therapeutic targets.This high-resolution analysis unveils cellular states, transcriptional programs, and signaling pathways driving tumor initiation, progression, and metastasis.

Importantly, scRNA-Seq identifies rare subpopulations of cancer cells with distinct gene expression signatures associated with aggressive phenotypes, therapy resistance, and metastatic potential. Sun et al. (32), conducted a comprehensive analysis of precancerous lesions in oral mucosa to understand their role in the initiation of OSCC. Their findings reveal altered gene expression in epithelial cells favoring OSCC initiation, along with distinct fibroblast, monocyte, and regulatory *T*-cell subclusters shaping the microenvironment. The study underscores the importance of immune regulation and spatial organization in OSCC initiation, offering valuable insights for targeted therapies. Furthermore, scRNA-Seq facilitates the delineation of intra-tumoral heterogeneity, elucidating clonal evolution dynamics and cellular interactions within the tumor ecosystem. These were evident by Choi et al. (33), his study employed scRNA-seq to elucidate the stepwise progression of head and neck squamous cell carcinoma (HNSCC) from normal to precancerous to metastatic tissues. Their study unveiled significant dysplasia and carcinoma *in situ* cells in precancerous tissues, identified crucial cell-cell interactions driving HNSCC progression, and highlighted dynamic alterations in T cell repertoires. These findings offer promising avenues for targeted interventions to manage HNSCC progression.

Integration of scRNA-Seq data with spatial transcriptomics techniques provides spatial context to cellular diversity, enabling the mapping of cell types and their interactions within the complex architecture of oral tumors. Song et al. (34) investigated the impact of chemotherapy on the tumor microenvironment (TME) in OSCC using single-cell transcriptome analysis. Their study revealed distinct cell types and molecular pathways associated with epithelial-mesenchymal transition (EMT), providing valuable insights into post-chemotherapy OSCC TME characteristics and potential therapeutic targets.

scRNA-Seq aids in the discovery of novel biomarkers and therapeutic targets by uncovering cell type-specific gene expression patterns and molecular vulnerabilities. Studies by Hsieh et al. (35) and Wang et al. (36) explored the influence of Candida albicans infection and CCR7 expression on OSCC development, respectively, using scRNA-seq technology. These studies identified specific cell clusters, oncogenic pathways, and altered gene expression profiles associated with OSCC progression and the tumor microenvironment, offering valuable insights for precision medicine applications and therapeutic interventions. Moreover, scRNA-Seq holds promise for guiding personalized treatment strategies by identifying predictive biomarkers of treatment response and resistance mechanisms. Overall, scRNA-Seq represents a powerful tool for deciphering the cellular and molecular landscape of oral cancer, with implications for understanding disease pathogenesis, improving patient stratification, and advancing precision oncology approaches.

### 3.5 Long non-coding RNA (lncRNA) profiling

Long non-coding RNAs (lncRNAs) have emerged as key players in the pathogenesis of oral cancer, and their profiling offers crucial insights into the regulatory mechanisms underlying tumorigenesis, progression, and metastasis. These transcripts, defined as RNA molecules longer than 200 nucleotides with limited protein-coding potential, participate in diverse cellular processes, including chromatin remodeling, transcriptional regulation, RNA splicing, and post-transcriptional gene expression modulation. In oral cancer, dysregulated expression of lncRNAs has been associated with oncogenic transformation, tumor growth, invasion, and metastasis. Furthermore, Jia et al. (37) investigated plasma lncRNA expression in oral squamous cell carcinoma (OSCC) for early diagnosis and staging. They identified 14 candidate lncRNAs, of which four (ENST00000412740, NR_131012, ENST00000588803, NR_038323) showed significant differences across healthy controls, oral premalignant lesions, and OSCC stages. Combining these lncRNAs enhanced diagnostic efficacy, particularly for advanced stages, suggesting their potential as promising biomarkers for OSCC diagnosis and staging.

Profiling lncRNAs in oral cancer tissues and cell lines enables the identification of aberrantly expressed transcripts that serve as diagnostic and prognostic markers. Arunkumar et al. (38) conducted expression profiling of lncRNAs in oral squamous cell carcinoma (OSCC) tissues and identified Linc-RoR as a prognostic biomarker. They found Linc-RoR to be significantly overexpressed in undifferentiated tumors associated with tobacco chewing, correlating with poor therapeutic response and tumor recurrence. This study suggests Linc-RoR as a potential prognostic marker in OSCC.

Moreover, functional characterization of dysregulated lncRNAs elucidates their roles in modulating key signaling pathways implicated in oral cancer progression, such as the epithelial-mesenchymal transition (EMT), Wnt/β-catenin, PI3 K/Akt, and Notch pathways. The study by Zhou et al., investigated the expression of lncRNAs in the transition from oral submucous fibrosis (OSF) to oral squamous cell carcinoma (OSCC). They identified 687 differentially expressed lncRNAs implicated in pathways related to inflammation, fibroelastic changes, and malignant progression. These findings underscore the importance of lncRNAs in OSF malignant development and highlight their potential as biomarkers or therapeutic targets. Add on lncRNA profiling facilitates the discovery of lncRNA-mRNA regulatory networks and their interactions with microRNAs (miRNAs) and proteins, contributing to a comprehensive understanding of the molecular mechanisms underlying oral cancer development and metastasis. Furthermore, lncRNA signatures derived from profiling studies hold promise for guiding personalized treatment strategies and identifying novel therapeutic targets for precision medicine approaches in oral cancer management. In summary, lncRNA profiling plays a pivotal role in deciphering the regulatory landscape of oral cancer and holds significant potential for advancing our understanding of the disease and improving clinical outcomes.

### 3.6 Small RNA profiling

Small RNAs, including microRNAs (miRNAs) and small interfering RNAs (siRNAs), play crucial roles in the post-transcriptional regulation of gene expression and are implicated in oral cancer progression. Profiling of small RNAs using techniques such as small RNA sequencing and microarrays provides insights into their dysregulation in oral cancer tissues, offering potential diagnostic biomarkers and therapeutic targets. Manikandan et al. (39) analyzed miRNA expression in oral squamous cell carcinoma (OSCC), identifying 46 differentially expressed miRNAs. They validated the downregulation of the let-7 family and miR-16, as well as the upregulation of miR-29b, miR-142-3p, miR-144, miR-203, miR-223, and miR-1275. Functional analysis implicated the involvement of the PI3K/Akt and p53 pathways in OSCC, highlighting miRNAs as potential diagnostic and prognostic biomarkers.

Furthermore, Punyadeera, et al. (40) developed a saliva-based microRNA signature for early oral cancer (OC) detection in oral potentially malignant disorders (OPMD). They identified and validated eight miRNAs showing high discriminative ability between OC and controls, as well as between OC and OPMD. This study suggests the clinical potential of salivary miRNAs for OC management. In addition, Pratama et al. (41) utilized a computational approach to authenticate differential gene expression in oral squamous cell carcinoma (OSCC) using machine learning applications. While the model effectively distinguished OSCC from other squamous cell carcinomas (SCCs) originating in different tissues, challenges arose in differentiating between SCCs from nearby sites. Nonetheless, potential diagnostic genes specific to OSCC, such as PRDM16 and DLC1, were identified, offering novel insights into OSCC diagnosis and treatment.

Overall, these studies underscore the importance of small RNA profiling in elucidating the regulatory networks underlying oral cancer pathogenesis and progression, offering potential biomarkers and therapeutic targets for further investigation and clinical translation.

**Figure 3:**
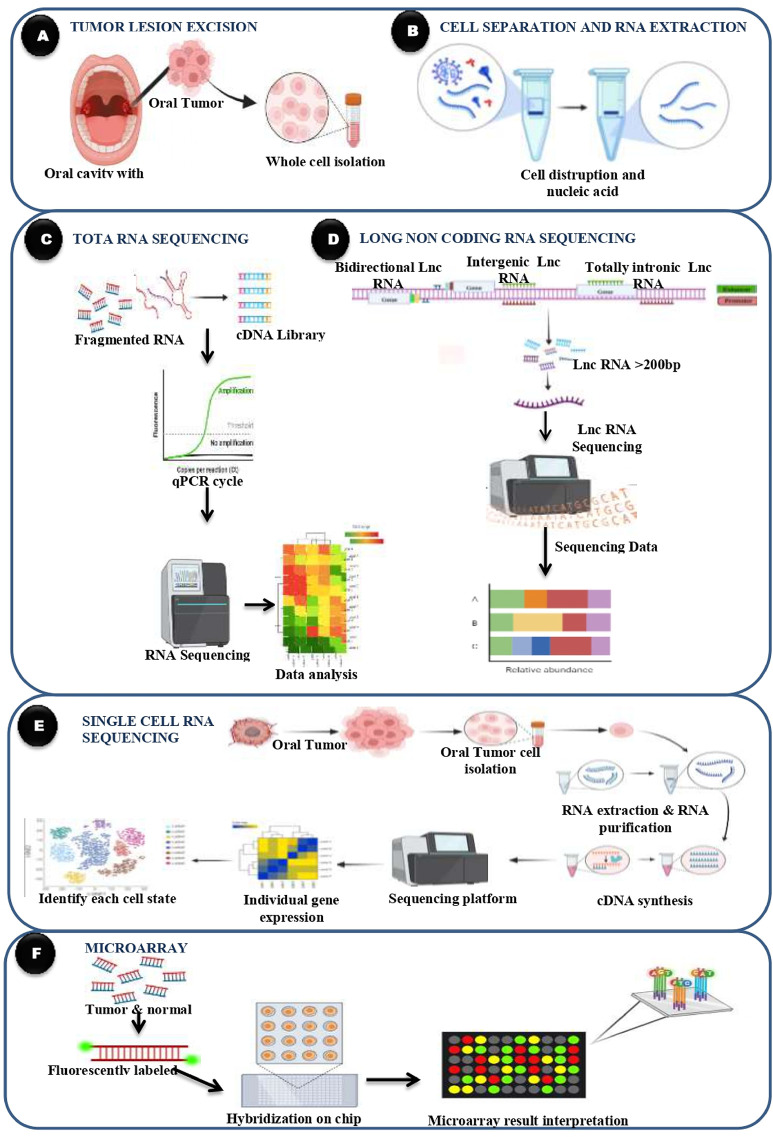
Transcriptomics technologies in oral cancer. **(A)** Tumor lesion excision involves obtaining tissue samples from the oral cavity containing the tumor, followed by whole cell isolation for molecular analysis. **(B)** The isolated cells undergo cell disruption and RNA extraction to purify nucleic acids, particularly RNA, for transcriptomic profiling. **(C)** In total RNA sequencing, the extracted RNA is fragmented and converted into a complementary DNA (cDNA) library. Quantitative PCR (qPCR) is used for amplification, followed by high-throughput sequencing and data analysis to determine global gene expression patterns. **(D)** Long non-coding RNA (lncRNA) sequencing focuses on identifying and quantifying lncRNAs typically longer than 200 base pairs including bidirectional, intergenic, and totally intronic lncRNAs, to assess their relative abundance and potential regulatory roles in cancer. **(E)** Single-cell RNA sequencing allows analysis at the level of individual cells. Tumor cells are isolated, and RNA is extracted and converted into cDNA. Sequencing and bioinformatics enable the identification of distinct cell states and gene expression profiles, revealing tumor heterogeneity. **(F)** Microarray analysis involves fluorescently labeling RNA from both tumor and normal samples. The labeled RNA is hybridized onto a chip, and the resulting signal intensities are used to compare gene expression levels across conditions, aiding in the interpretation of transcriptomic differences in oral cancer.

By employing these transcriptomic profiling techniques, researchers can gain a comprehensive understanding of the molecular landscape of oral cancer, identify novel biomarkers for early detection and prognosis, and uncover potential therapeutic targets for precision medicine approaches (Figure 3). These techniques continue to advance our knowledge of oral cancer biology and hold promise for improving patient outcomes through personalized treatment strategies. Transcriptomic profiling techniques in oral cancer encompass various methods aimed at comprehensively analyzing gene expression patterns and regulatory networks within oral cancer tissues. RNA sequencing (RNA-Seq) provides a comprehensive view of the transcriptome by quantifying gene expression levels and detecting alternative splicing events, identifying differentially expressed genes and novel transcripts. Microarray analysis enables the simultaneous measurement of thousands of genes’ expression levels, facilitating the discovery of potential biomarkers and therapeutic targets. Single-cell RNA sequencing (scRNA-Seq) offers insights into cellular heterogeneity within oral tumors, identifying rare cell types and transcriptional states associated with disease progression. Profiling of long non-coding RNAs (lncRNAs) and small RNAs, including microRNAs (miRNAs) and small interfering RNAs (siRNAs), provides further understanding of their roles in oral cancer pathogenesis, offering potential biomarkers and therapeutic targets. These transcriptomic profiling techniques collectively contribute to a comprehensive understanding of oral cancer biology, facilitating the identification of novel biomarkers for early detection, prognosis, and therapeutic targets for precision medicine approaches.

## 4 Differential gene expression analysis in oral cancer

**Figure 4:**
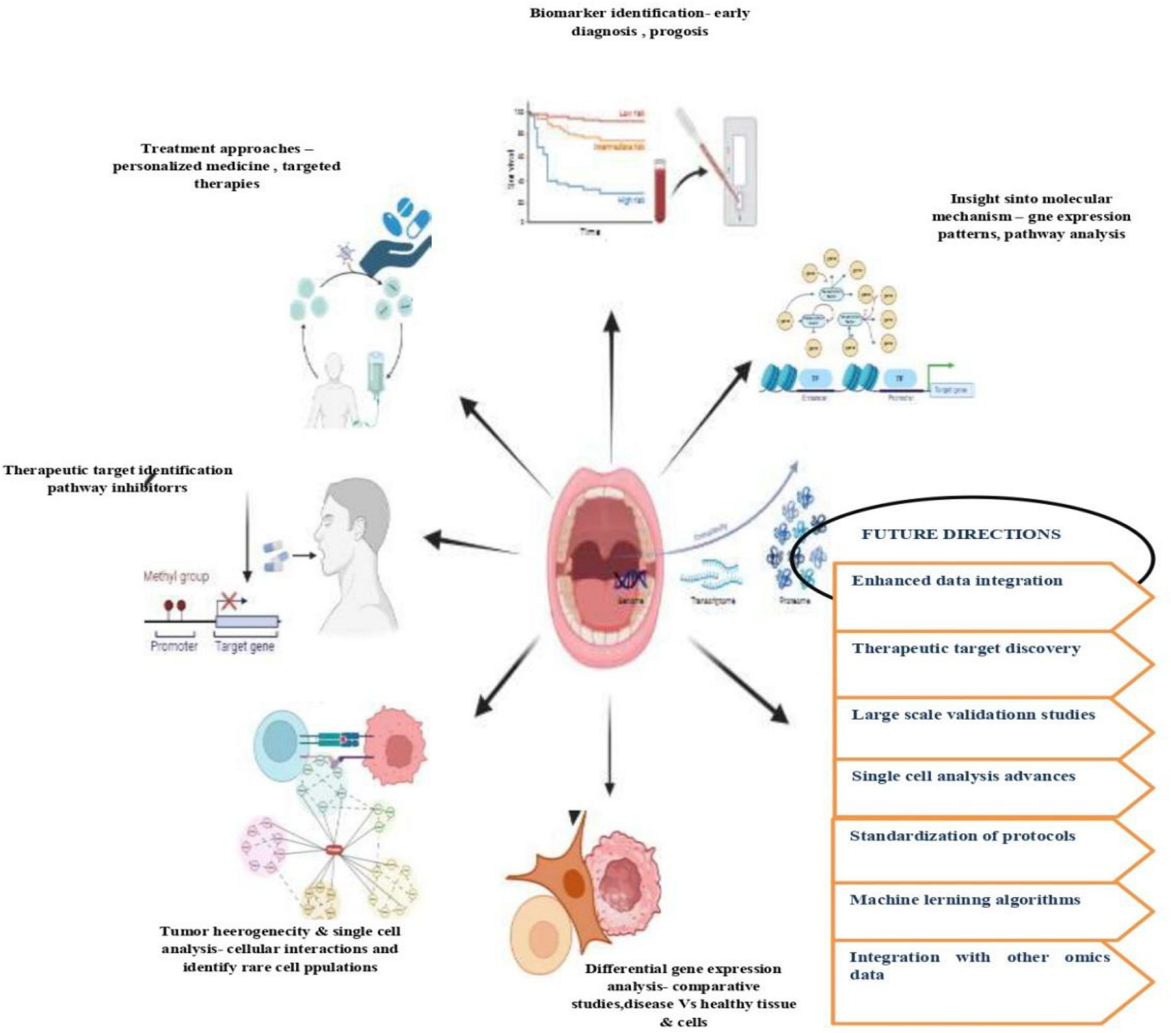
Transcriptomics application and future directions in oral cancer.

Differential gene expression analysis in oral cancer is a pivotal area of research that focuses on identifying genes with altered expression patterns compared to normal oral tissues. Through transcriptomic profiling techniques such as RNA sequencing and microarray analysis, researchers can quantify gene expression levels and pinpoint genes that are upregulated or downregulated in oral cancer (Figure 4). This approach provides invaluable insights into the molecular mechanisms driving tumorigenesis in the oral cavity. Ye et al. (42) conducted a transcriptomic dissection of tongue squamous cell carcinoma (TSCC), revealing specific gene expression signatures distinguishing tumor tissues from normal counterparts. Upregulated genes included MMP1, MMP10, IL8, and KRT17, while downregulated genes included KRT4, MAL, and CRNN. Functional analysis highlighted alterations in biological processes such as collagen catabolism, NF-kappaB signaling, and extracellular matrix organization in OTSCC, offering potential diagnostic and screening tools. Numerous dysregulated genes have been discovered through this analysis, shedding light on key biological processes implicated in oral cancer progression. An exemplary study supporting the significance of transcriptomic analyses in oral cancer is the research conducted by Zhang et al. (43). This study utilized transcriptomic analysis to dissect the gene expression patterns in tongue squamous cell carcinoma (TSCC), a common type of oral cancer. By elucidating the transcriptomic landscape, the researchers identified dysregulated genes and pathways involved in TSCC pathogenesis, providing crucial insights into key molecular mechanisms underlying tumor initiation and progression.

By elucidating the landscape of differential gene expression, researchers gain a deeper understanding of the molecular heterogeneity of oral tumors, identifying distinct subtypes with unique gene expression signatures.One notable study by Singh et al. (16) aimed to differentiate well-differentiated (WD) from moderately/poorly differentiated (MD/PD) oral squamous cell carcinoma (OSCC) by identifying gene signatures and novel RNA isoforms. Through various sequencing techniques, the researchers revealed differentially expressed transcripts and fusion transcripts, highlighting potential cancer pathways and therapeutic targets.In summary, differential gene expression analysis in oral cancer is instrumental in unraveling the molecular complexities of the disease and guiding the development of targeted therapeutic interventions, ultimately improving patient outcomes in the management of this challenging condition.

## 5 Insights into molecular mechanisms

Transcriptomic analyses offer valuable insights into the molecular mechanisms driving oral cancer pathogenesis. Zhang et al. (16) examined gene expression patterns in tongue squamous cell carcinoma (TSCC), uncovering dysregulated genes and pathways involved in tumor initiation and progression, including alterations in cell cycle regulation, apoptosis, DNA repair, EMT, and angiogenesis. Pannone et al. (44) utilized next-generation sequencing to reveal molecular signatures and pathways associated with OSCC tumorigenesis and metastasis. These studies highlight dysregulated pathways such as cell proliferation, apoptosis, and EMT, contributing to OSCC development.

Furthermore, Neetu Singh et al. (45) distinguished well-differentiated (WD) from moderately/poorly differentiated (MD/PD) OSCC subtypes, identifying gene signatures and novel RNA isoforms linked to potential cancer pathways and therapeutic targets. Integrating transcriptomic data with other omics approaches and functional studies enables the identification of key transcription factors, signaling molecules, and epigenetic modifications driving oncogenic transformation and tumor aggressiveness in oral cancer. Ultimately, these insights pave the way for developing targeted therapies and personalized treatment strategies aimed at disrupting specific molecular pathways and improving patient outcomes.

## 6 Identification of biomarkers

Transcriptomic analysis is instrumental in identifying biomarkers for oral cancer, facilitating early detection, prognosis prediction, treatment stratification, and disease monitoring. Studies like those by Simon A. Fox et al. (46) have utilized advanced algorithms to identify novel biomarkers, such as MAMDC2, SYNPO2, and ARMH4, associated with oral squamous cell carcinoma (OSCC) tumorigenesis and metastasis. These biomarkers (Table 2) aid in discriminating between tumor tissue and surgical margins, improving detection methods for residual cancer cells and enhancing patient care.

**Table 2:** Oral cancer biomarker identification through transcriptomics.

S.no.	Biomarker	Description	Reference
1	miR-21	Overexpressed in oral cancer tissues; potential diagnostic and prognostic marker	(48)
2	miR-31	Upregulated in oral cancer; associated with metastasis and poor prognosis	
3	miR-375	Downregulated in oral cancer; potential tumor suppressor; linked to aggressive behavior	
4	TP53	Frequently mutated in oral cancer; associated with tumor progression and poor prognosis	(55)
5	EGFR	Overexpression correlated with oral cancer development and aggressiveness	
6	CDKN2A	Frequently altered in oral cancer; involved in cell cycle regulation and tumor suppression	
7	SOX2	Overexpression associated with oral cancer progression and metastasis	(56)
8	NOTCH1	Dysregulated in oral cancer; implicated in cell proliferation and differentiation	
9	MMP9	Elevated expression linked to invasion and metastasis in oral cancer	
10	BIRC5 (Survivin)	Overexpression associated with oral cancer progression; potential therapeutic target	(11)
11	FAM83A	Upregulated in oral cancer; involved in cell proliferation and metastasis	
12	CCND1 (Cyclin D1)	Overexpression correlated with oral cancer development and progression	
13	IL6	Elevated levels associated with oral cancer aggressiveness; promotes tumor growth and metastasis	(57)
14	PTGS2 (COX-2)	Upregulated in oral cancer; implicated in inflammation and tumor progression	
15	VEGFA	Overexpression associated with angiogenesis and poor prognosis in oral cancer	
16	MALAT1	Upregulated in oral cancer; involved in tumor proliferation, invasion, and metastasis	(58)
17	HOTAIR	Overexpression correlated with oral cancer progression and metastasis	
18	MEG3	Downregulated in oral cancer; functions as a tumor suppressor; inhibits cell proliferation and invasion	

Allan Radaic et al. (47) further explore biological biomarkers of oral cancer, highlighting the multifaceted nature of biomarker discovery in this context. Additionally, the investigation of microRNAs (miRNAs) and circular RNAs (circRNAs) as biomarkers, as demonstrated in studies by Palaia et al. (48), reveals their potential for diagnosing OSCC with higher sensitivity and specificity compared to traditional markers. CircRNAs, in particular, emerge as promising diagnostic biomarkers due to their ability to modulate gene expression and influence critical biological processes implicated in oral cancer progression. These findings collectively underscore the significance of transcriptomic analyses in uncovering novel biomarkers and advancing diagnostic approaches for oral cancer management, ultimately improving patient outcomes and addressing the clinical need for more effective diagnostic methods.

## 7 Tumor heterogeneity and single-cell analysis

Tumoral heterogeneity has emerged as a critical concern in cancer research, particularly in tailoring personalized cancer treatments. Oral squamous cell carcinoma (OSCC) poses a unique challenge due to its microenvironment within the oral cavity, influenced by factors such as diverse tissue compositions, microbial flora, various carcinogenic exposures, potentially malignant conditions, epithelial turnover rates, saliva, and proximity to external factors. This complex microenvironment has the capacity to shape the development of numerous oral pathologies, including OSCC.

Transcriptomic analysis, particularly through single-cell RNA sequencing (scRNA-seq), offers a powerful approach to explore the intricate tumor heterogeneity in oral cancer. Single-cell RNA sequencing (scRNA-Seq) allows for the characterization of individual cells within oral tumors, identifying rare cell types, tumor subtypes, and transcriptional states associated with disease progression. Studies like that of Chen et al. (49) delve deep into the molecular landscape of oral squamous cell carcinoma (OSCC), unraveling distinct cellular subpopulations within tumors and shedding light on their transcriptional signatures linked to various tumor cell states, including stemness, proliferation, and immune evasion. Furthermore, investigations into the intratumoral landscape of infiltrated *T*-cell subpopulations in OSCC, as demonstrated by Chen et al. (49), reveal the complexity of the tumor microenvironment and provide insights into potential therapeutic targets for immunosuppression.

Moreover, as highlighted by Hao Song et al. (34), scRNA-seq facilitates the analysis of changes in the tumor immune microenvironment post-chemotherapy, uncovering distinct inflammatory cytokine expression patterns and epithelial-mesenchymal transition (EMT) dynamics. These findings collectively deepen our understanding of OSCC biology, informing personalized treatment strategies and the development of targeted therapies tailored to specific cellular contexts within the tumor. Considering the multifaceted nature of the oral tumor microenvironment, transcriptomic approaches like scRNA-seq offer invaluable insights into tumor heterogeneity, paving the way for more effective therapeutic interventions and improved patient outcomes.

## 8 Prediction of treatment response

Transcriptomic analysis has emerged as a promising tool for predicting treatment response in oral cancer patients. Studies such as that by Tada et al. (50) and Kiong et al. (51) have demonstrated the utility of transcriptomic profiling in identifying molecular signatures associated with chemotherapy and immunotherapy response, respectively. By analyzing gene expression patterns in pre-treatment tumor samples, these studies identified transcriptomic signatures indicative of treatment sensitivity or resistance. Moreover, the predictive capacity of these signatures was validated in independent cohorts of patients, underscoring their potential clinical relevance in guiding treatment decisions and improving patient outcomes.

Additionally, Brown et al. (52) elucidated molecular signatures associated with chemotherapy response in oral squamous cell carcinoma (OSCC), highlighting the role of genes involved in drug metabolism, DNA repair, and cell cycle regulation. Collectively, these findings emphasize the importance of transcriptomic analysis in personalized medicine approaches for oral cancer, enabling tailored treatment strategies based on individual patient profiles and optimizing therapeutic efficacy while minimizing adverse effects. By predicting treatment response through transcriptomic analysis, clinicians can make informed decisions, monitor treatment progress, and identify potential resistance mechanisms, ultimately enhancing patient care and outcomes in oral cancer management.

## 9 Identification of therapeutic targets

Transcriptomic analysis serves as a crucial tool in identifying therapeutic targets and biomarkers for oral squamous cell carcinoma (OSCC), contributing to precision medicine approaches in cancer treatment (Table 3). Studies by Gollapalli et al. (53), highlight the significance of transcriptomic profiling in uncovering dysregulated genes and pathways associated with OSCC tumorigenesis. Through integration of transcriptomic and genomic data, these studies identify novel therapeutic targets with druggable properties, providing insights into potential treatment avenues for OSCC patients. Furthermore, research by Moazam (54) emphasizes the utility of transcriptomic analysis in identifying candidate genes and pathways dysregulated in OSCC, offering opportunities for precision medicine interventions. Their findings demonstrate the clinical relevance of identified targets in OSCC patient cohorts, paving the way for personalized treatment strategies tailored to individual patients. Additionally, investigation focus on transcriptomic biomarker signatures for discriminating oral cancer surgical margins, highlighting their potential to improve surgical outcomes by reducing the risk of tumor recurrence. Together, these studies underscore the importance of transcriptomic analysis in advancing our understanding of OSCC pathogenesis and guiding personalized therapeutic interventions and surgical decision-making, ultimately aiming to improve patient outcomes and quality of life.

**Table 3:** Novel discoveries by transcriptomics in oral cancer.

S.no.	Study title	Gene name(S)	Reference
1	Transcriptomic Profiling Reveals Dysregulated Pathways in Oral Squamous Cell Carcinoma	TP53, EGFR, CDKN2A	(54)
2	Identification of Key Gene Signatures Associated with Oral Cancer Progression	SOX2, NOTCH1, MMP9	(57)
3	Integrative Analysis of Transcriptomic Data Unveils Potential Biomarkers for Oral Cancer Diagnosis	BIRC5, FAM83A, CCND1	(58)
4	Network-based Transcriptomic Analysis Identifies Hub Genes in Oral Squamous Cell Carcinoma	IL6, PTGS2, VEGFA	(49)
5	Long Non-coding RNAs as Novel Regulators in Oral Cancer Transcriptome	MALAT1, HOTAIR, MEG3	(49)
6	RNA Sequencing Reveals Novel Fusion Genes in Oral Squamous Cell Carcinoma	MYB-NFIB, BCR-ABL, EWSR1-FLI1	(34)
7	Identification of Circulating miRNAs as Diagnostic Biomarkers for Oral Cancer	miR-21, miR-31, miR-375	(59)
8	Genome-wide Analysis Uncovers Epigenetic Alterations in Oral Cancer	DNMT1, HDAC1, H3K27me3	(48)
9	Differential Expression of lncRNAs in Oral Squamous Cell Carcinoma Revealed by RNA-seq	HOTAIR, MALAT1, H19	(52)
10	Transcriptomic Profiling Identifies Novel Immunotherapy Targets in Oral Cancer	PD-L1, CTLA-4, TIGIT	
11	Longitudinal Transcriptomic Analysis Reveals Dynamic Gene Expression Changes during Oral Cancer Progression	E-cadherin, Slug, Vimentin	(50)
12	Identification of Fusion Genes in Oral Squamous Cell Carcinoma Using Transcriptomic Approaches	MYB-NFIB, PAX3-FOXO1, EWSR1-FLI1	(55)

## 10 Challenges and future directions

Despite significant advancements, transcriptomic research in oral cancer faces several challenges. These include data analysis complexity, sample heterogeneity, and the need for functional validation of candidate biomarkers and therapeutic targets. Additionally, integrating transcriptomic data with other omics approaches and clinical parameters poses computational and logistical challenges. Future directions in transcriptomic research in oral cancer include the development of innovative computational tools and experimental models to address these challenges, as well as the implementation of multi-omics approaches to gain a more comprehensive understanding of the molecular landscape of the disease. Standardized procedures in RNA extraction and sequencing alongside standardized data analysis methods need implementation to achieve study consistency. A holistic disease mechanism emerges when researchers integrate proteomic and genomic data and epigenomic information with transcriptomic biomarker research to produce more accurate biomarkers. Clinical evidence can be established through evaluations based on cells and animal subjects along with organoids derived from patients to validate transcriptomic results. Clinical trials and real-life patient information must be used prospectively to establish how well transcriptomic markers function in predicting both treatment outcomes and patient clinical results. The promotion of transparency requires collaborative work between organizations that share data and form global research partnerships for cross-validation of results. Longitudinal studies are needed to monitor treatment response and disease progression over time, and collaborative efforts are essential to validate transcriptomic findings in large patient cohorts. Ultimately, overcoming these challenges and advancing transcriptomic research in oral cancer will pave the way for the development of more effective targeted therapies and personalized treatment approaches, leading to improved outcomes for patients with this devastating disease.

## 11 Conclusions

The integration of transcriptomics into oral cancer research has significantly advanced our understanding of the disease at a molecular level. Through various high-throughput sequencing and profiling techniques, researchers can now identify crucial biomarkers, explore differential gene expression patterns, and understand the complexities of tumor heterogeneity. These insights are essential for developing targeted therapies and improving patient outcomes. However, challenges such as data integration, standardization of protocols, and the need for large-scale studies remain. Future research should focus on overcoming these challenges to fully realize the potential of transcriptomics in transforming oral cancer diagnosis, prognosis, and treatment.
